# Understanding the emotion of shame in transgender individuals – some insight from Kafka

**DOI:** 10.1186/s40504-018-0085-y

**Published:** 2018-10-01

**Authors:** Simona Giordano

**Affiliations:** Centre for Social Ethics and Policy, The School of Law, University of Manchester, Williamson Building, Oxford Road, Manchester, M13 9PL England

## Abstract

Both clinical literature and biographical accounts suggest that many transgender individuals experience shame or have experienced shame at some point in their life for reasons related to their gender identity. In clinical psychology, at least until the 1960s, shame has not received much attention; focus was on guilt and shame was regarded mainly as a ‘by-product’ of guilt. From the 1960s shame has been identified as an emotion not necessarily related to guilt and with unique features, and has been studied in connection with a number of situations, such as domestic abuse, trauma, illness, and sexual orientation. However shame has been studied less in connection with gender variance. Shame has however intrigued philosophers, writers and artists for a very long time. Yet, the importance of the contribution of various disciplines to the understanding of the experience of shame in vulnerable individuals has been overlooked. This paper attempts to explore the meaning of shame for transgender individuals, by making reference not only to clinical studies, but also to artworks and literary novels. Franz Kafka, named “the poet of shame” is particularly salient to the analysis of shame, and some of his works will enable us to shed light on the complexities of the experience of shame in transgender individuals which may defy clinical observation.

## Introduction

*What is ‘shame’?*

*Shame is a form of sadness*

(A child)

Biographical accounts by transgender individuals are replete with references to shame and shaming experiences (ranging from feeling ashamed about disclosing one’s experienced gender identity, to feeling ashamed of one’s natal sex, to shaming experiences of being verbally or physically abused, bullied at school or discriminated in the work place). Not only biographical accounts, but also published research evidences a correlation between being transgender and experiencing shame (see “Methodology and de-limitations” section). However, with some exception to be found in queer literature (Munt, 2000; Probyn, 2000; Probyn, 2004a; Probyn, 2005; Sedgwick, 2003), there is paucity of systematic studies of the experience of shame in transgender individuals. For example, what triggers shame in the particular case of transgender individuals at various stages of their life and of their journey towards disclosure and transition, how shame is experienced in various context (in the family, at school for younger transgender people, in the work place, in the group of peers, in intimate relationships), what coping mechanisms are typically implemented, what the impact of shame is likely to be on the psychological welfare of transgender individuals, remain scarcely explored.

Clinical psychology has started being interested in shame as a unique emotion only relatively recently, approximately since the 1960s, and shame, as we shall see later in greater detail, has been studied mainly in connection with trauma, domestic abuse, illness and sexual orientation. Existing studies show that persistent shame is associated with psychological trauma, self-destructive behaviour and suicide (see “A brief account of shame and its psychological impact” section). Given that suicidality, suicide attempts and self-harming behaviour are more prevalent among transgender people than in cisgender people, it would be important to understand whether shame plays any part in this. This paper will not provide an empirical analysis of shame in transgender individuals; instead it will offer a philosophical analysis of the emotion of shame, a reflection on what it may mean to experience shame, how shame can manifest itself and, ultimately, how shame can be understood and dealt with.

To this purpose, I will use a number of sources from various disciplines (particularly narrative,^1^ philosophy, and figurative arts). This choice is inspired by the work of Carl Goldberg, who, in a seminal work on shame, pointed out how these other disciplines can tell us something important about shame, and how the contribution of these disciplines to the psychological understanding of shame has often been overlooked (Goldberg, 1991a).

Indeed shame has intrigued philosophers, anthropologists, writers (Strandberg, 2004, Nixon, 2009) and artists for a long time - see for example *The art of shame* by Marcel Duchamp (around 1912)^2^ and *Shame and Desolation* by Alessandra Favetto (1968-)^3^, and later for more examples. Children’s literature is also populated by ashamed heroes – usually because of their diversity (The Ugly Duckling, the Hunchback of Notre Dame and many others). This article will not offer an analysis of the literary constructions of shame; rather, by using various sources in tandem, it hopes to shed light on aspects of shame that may defy clinical observation.

It will be noted that questions about our gender are in essence questions about who we are as persons, and thus being ashamed about our gender is also an attack on our status as persons. Gender is an intimate and private matter, and at the same time it has an obvious public dimension. This, as will be explained, renders people who have atypical gender identities particularly exposed to shaming experiences and to the associated risks.

This paper will conclude with some recommendations on how to tackle shame in transgender individuals. These recommendations are both clinical and socio-political. It will be argued that without a proper and nuanced understanding of shame, it is difficult to understand the experience of gender variance, and the coping mechanisms that people may implement to minimise the damaging effects of shame. Attempting to focus on shame brings to light the fact that many of the predicaments usually associated with gender variance have primary social determinants, and are not primarily intra-psychic; in other words, shame is a social issue, not just a psychological issue. This means that in order to address shame, and to prevent the harms associated with it, it is not sufficient to work with individuals within a clinical setting: it is necessary to also reflect and address, with appropriate social and public policy interventions, the social determinants of shame, and to reflect on the goals of therapeutic intervention.

A terminological clarification: I will use the word transgender in its wider sense, borrowing Susan Stryker’s definition: “an umbrella term that refers to all identities and practices that cross over, cut across, move between, or otherwise queer socially consgender binari/gender binaries” (Stryker, 1994). So I will not differentiate between transgender and transsexual people based on whether they have received medical or surgical treatments; I will also use the term gender variance to refer broadly to the experience of belonging to a gender minority.

## Methodology and de-limitations

A research conducted on JSTOR databases of the terms “transgender” and “shame”, under the following disciplines: *Feminism and Women Studies; Film Studies; Health Policy; Health Sciences; Law; Management and Organizational Behavior; Philosophy; Political Sciences; Psychology; Public Health; Sociology* from 2007 to 2017 produced 423 entries. Virtually all these publications highlight that shame and guilt are common emotions among LGBT people. A simple google search will also provide significant anecdotal evidence of the correlation between shame and gender variance.

It ought to be noted, however, that, with a few exceptions,^4^ a large part of the literature on shame in LGBT people does not focus specifically on *transgender individuals* (Cianciotto & Cahill, 2012; Nyong’o, 2009; Warren, 2010; Bender, 2015; Anderson, 2009a; Buttaro, 2012a; Wallace & Russell, 2013; Kaufman & Raphael, 1996; Wells & Downing, 2003b). This, it may be believed, should not be of concern; because as “the T” of the LGBT groups, transgender individuals are among those to whom the studies refer so they are included in the subjects being studied anyway. However, this is not always the case, because in many of these studies the empirical research on people’s coping mechanism, and on the determinants and consequences of shame, is focused specifically on homosexual people. The results of those studies may not therefore be straightforwardly applied to transgender individuals.

This article uses the methodology that is typical of the analytical investigations in applied ethics: literature review and conceptual analysis. A review of the psychology literature has been performed, using the above mentioned databases and key terms, and the entries have been carefully read by the author. In addition to clinical studies, I have used other sources: biographical accounts (both in the form of published autobiographies and personal narrations), artwork, philosophical work, and literary sources (including children’s literature). Although this work is thus interdisciplinary, in that it uses sources from various disciplines, the reader will not find a discernible interdisciplinary methodology.

Half way through the paper we will pause on Franz Kafka (who was named by a commentator “poet of shame”) (Friedlander, 2013), particularly on his *Trial* and *Metamorphosis*; these novels are selected because provide extraordinarily insightful and penetrating accounts of shame and will help us to understand the insidious nature of shame. Importantly, the literary texts are not employed to substantiate a pre-existing hypothesis and are not used to provide clinical evidence. The purpose of this work is to enhance the understanding of shame, and the sources selected (including the scientific studies) are used as they tell something important and interesting about this emotion.

The sources are delimited to the Western culture and mainly to the period ranging from the second half of the 1900s to the end of 2017. An anthropological comparative review of the literature goes beyond the remit of this paper. For a brief overview of cross-cultural studies of gender identity see references provided (Giordano, 2013a; Roen, 2006).

Another delimitation (and hence limitation) of this article is that what is argued here is most likely to apply to *white* transgender people, particularly adults or adolescents. As various authors suggest, the experience of shame is likely to be different in different ethnic groups (Buttaro, 2012b; Ramirez-Valles, 2011; Ramirez-Valles et al., 2010; Spalek, 2008a; Spalek, 2008b). The experience of shame, thus, may pitch to different recipients and take different forms and hence the delimitation chosen here.

These geographic and ethnic delimitations represent clear substantial (not only methodological) limits: however, it is likely that any discourse on gender identity and diversity will encounter similar limitations. Gender is a function of social, political, historical, cultural, economic and religious factors, as well as individual factors (Giordano, 2013b). The experiences associated with gender variance, and consequently shame and guilt, are thus also likely to differ significantly in different contexts. However, this should not prevent us from seeking and valuing a better understanding of the experiences that are most likely to occur within specific communities.

## A brief account of shame and its psychological impact

This section provides a brief account of shame, as is currently treated in clinical psychology. This account relies particularly on the works of Silvan Tomkins, who has been one of the first psychologists to differentiate shame from guilt, Tangney and Probyn. The works on shame by Carl Goldberg provided the impetus for the use of sources from a range of disciplines (Goldberg, 1991b).

Shame and guilt are regarded as ‘self-conscious’ emotions (Tracy et al., 2007) in clinical psychology. Traditionally, at least in the psychoanalytic/psychodynamic tradition, guilt has been regarded as the most important between the two, because of its moral nature; shame has been either overlooked or incorporated in the notion of guilt.

Since the 1960s,^5^ shame has been regarded as an emotion distinct from guilt (Kim et al., 2011a; Tangney & Dearing, 2002a), and has been studied in connection with a number of situations (domestic abuse, trauma, illness, sexual orientation)^6^ (Schooler et al., 2005; Flood Aakvaag et al., 2014; Martins et al., 2016); however, shame has been studied less in connection with gender identity.

Guilt, it is argued, is “concerned with a negative evaluation of a specific behaviour” (Muris et al., 2015a). Shame, instead, “pertains to a negative evaluation of the global self” (“*I* did that wrong” as opposed to “I did *that* wrong”).

“Feelings of shame involve a painful focus on the self—‘*I* am a bad person’—whereas feelings of guilt involve a focus on a specific behavior—‘I did a *bad thing*’” (Tangney et al., 2014a).

Goldberg defines shame as “feeling ridiculous, embarrassed, humiliated, chagrined, mortified, shy, reticent, painfully self-conscious, inferior, and inadequate” (Goldberg, 1991a). And writes: “The shame-bound person has learned from others and now accuses himself of the ‘crime’ of being surplus, unwanted, and worthless” (Goldberg, 1991c).

Shame, according to some psychologists, makes people want to withdraw, hide, run away, become small and cover up (Tangney et al., 2014b; Probyn, 2004b).

Indeed many representations of shame in figurative arts portray the ashamed subjects in the act of covering up. A famous example is Masaccio’s (1401–1428) *Cacciata dei progenitori dall’Eden (The Expulsion from the Garden of Eden)*^7^. Adam is represented in the act of covering his eyes, and Eve in the act of covering her genitals. Both appear in great distress. Without pretending to propose any biblical hermeneutics, it may be worth also mentioning the episode known as “the drunkness of Noah”, subject of much interpretative work (Pedone, 2012) and of many representations (Michelangelo’s painting in the Sistine Chapel^8^). The Genesis narrates the story of Ham, one of Noah’s sons, who finds his father undressed and overcome by the effects of alcohol. He looks for his brothers, Shem and Japheth: *“And Shem and Japheth took a robe, and putting it on their backs went in with their faces turned away, and put it over their father so that they might not see him unclothed”*. They cover their father and walk away from the scene to overcome their shame (Genesis, 20–23).^9^ See also *La Pudicizia* by Antonio Corradini (1688–1752)^10^. Mentioned earlier, the photographer Alessandra Favetto illustrates shame as a woman nearly fully covered by a veil.^2^

According to studies in clinical psychology, people who feel *guilty* often want *to do something about it*: apologise, confess, or attempt to repair the damage done: they do not necessarily hide. Guilt is thus regarded as more ‘prosocial’ and less maladaptive than shame.

Although both shame and guilt correlate with psychological suffering, particularly with a wide range of anxiety disorders (Muris et al., 2015b), shame is thus usually considered more perilous than guilt. One piece of research found that “shame (but not guilt) remained significantly associated with higher levels of anxiety proneness and anxiety symptoms” (Muris et al., 2015c); another found that “shame exhibited a significantly stronger association with depressive symptoms compared to guilt” (Tangney & Dearing, 2002a; Kim et al., 2011b; Tangney, 1995).

According to one interpretation, this is because guilt, in that it prompts reparation, rather than passivity and rumination, may counter the likelihood of at least some anxiety disorders, such as depression. Pattison also notes that “shame has far more serious and pervasive moral implications than is often recognised in a context where most people think of guilt as the main element that is involved” (Pattison, 2003a) in psychopathology.

Shame is thus usually regarded as more clinically significant than guilt, as it is potentially more harmful than guilt.

Not everyone accepts that shame and guilt can be separated in the way highlighted here (Silfver, 2007; Pattison, 2003b). As we shall see in “Transgender stories: am I feeling guilt or shame?” section, it is particularly difficult to differentiate shame and guilt in the personal accounts of transgender individuals. Perhaps a rigid distinction between shame and guilt is arbitrary and artificial. However, the effort to separate conceptually these two emotions has enabled researchers to identify the social determinants of shame, and this makes it possible to put into focus the social dimension of the predicaments that often afflict transgender individuals.

## Shameful genders: transphobia and shame

In *Stone Butch Blues*, an early and powerful semi auto-biographical book, Lesley Feinberg narrates the horrific event of Jess being stripped naked by neighborhood children.

“I was filled with horror. I couldn’t make them stop. *The shame* of being half-naked before them—the important half—took all the steam out of me” (Feinberg, 2003) (my emphasis).

Then Jess is tied up and locked in a neighbor’s coal bin.

According to the House of Commons Women and Equalities Committee Transgender Equality First Report of Session 2015–16, experiences of bullying and transphobia are still common in the UK. One research participant reports: “being harassed, spat at, run over in one instance, sexually assaulted, beaten, having dog faeces thrown at me, stones thrown at me, head-butted, even my carer was physically assaulted for associating with me because I am transgendered” (House of Commons Women and Equalities Committee Transgender Equality First Report of Session, 2015). In its response to the Report, the UK Government notes: “We know that transgender people face continuing transphobia, increased mental health issues, discrimination in the provision of public and private services and bullying in our schools” (Minister for Women and Equalities by Command of Her Majesty, 2016). According to a 2016 report, reported hate crimes towards transgender people have risen by 41% in the year 2015–16 compared to the year 2014–15 (Corcoran & Smith, 2016).

Unsurprisingly hate crime is associated with a number of psychological problems (House of Commons Women and Equalities Committee Transgender Equality First Report of Session, 2015). However, interestingly some clinical studies suggest that such hostility also has a *shaming* effect. In fact, studies on shame found that *the determinants* of shame are likely to be discrimination, threats and similar forms of *social rejection* (Kim et al., 2011b; Tangney & Dearing, 2002b).

In a commentary of Feinberg’s book cited earlier, Stafford writes that acts of humiliation and violence such as those narrated by Jess in the book are a process of divesting the victims of their humanity; she calls this a process of *shaming* (Stafford, 2012*)*.

Similarly Sandra Anderson writes: “many [LGBT individuals] have internalised hostile treatment […] resulting in guilt, *shame*, low self-esteem, depression and substance abuse” (Anderson, 2009b) (my emphasis). Eli Clare offers an interesting account of how shame may be the root of many predicaments associated with gender variance, and makes an intriguing comparison between the stigma associated with disability and the stigma associated with gender variance (Clare, 2013).

Wells Downing and Hansens report that “[…] internalized homophobia places gays and lesbians at higher risk for developing *a shame-based identity*, especially when considering the lack of traditional supports available to cope with their disparaged ‘differentness’ and marginalized status” (Wells and Downing Hansen, n.d.).

In the next section, we will see how shame is thought to correlate with self-destructive behaviour and will discuss what this can tell us about self-destructive behaviour in transgender individuals.

## Shame and self-destructive behaviour in transgender individuals

We have seen earlier in “A brief account of shame and its psychological impact” section that, according to a number of clinical studies, shame tends to be maladaptive and is associated with withdrawal, psychological suffering, a wide range of anxiety disorders (Muris et al., 2015b) and depression (Tangney & Dearing, 2002a; Muris et al., 2015c; Kim et al., 2011b; Tangney, 1995).

Guilt is presented as an emotion directed to action(s), and as an emotion that usually prompts prosocial and reparative action; shame is instead presented as an emotion directed to the whole self, as an emotion that usually prompts withdrawal and hiding, and which is painfully experienced as beyond reparation. For these reasons shame is regarded as more dangerous than guilt (see earlier).

Goldberg pointed out that shame does not only prompt hiding and withdrawal, but also self-destructive behaviour, even suicide (Goldberg, 1991d).

A number of studies show that self-destructive behaviours, suicide attempts and suicide rates are much higher among transgender people than in the general population (Mcdermott et al., 2008a; Rivers, 2000). If Goldberg is right, it could mean that such high prevalence of self-destructive behaviour may be the result not of gender dysphoria, but of the discrimination, abuse and hatred of which gender minorities are still victim in many countries, including England (House of Commons Women and Equalities Committee, 2016).

Research on homosexual/lesbian people has shown a clear link between homophobia and self-destructive behaviours (Mcdermott et al., 2008b; King et al., 2008).

In one study of lesbian women, it was found that shame (predictably) decreased as social integration increased (Wells and Downing, 2003a).

As mentioned earlier, in most of the studies that examine shame in LGBT, the research participants are lesbian, gay and bisexual people, and not transgender individuals. It is therefore not clear how the experience of shame in transgender individuals correlates to self-destructive behaviour.

However, it is not implausible to postulate a link between transphobia and self-destructive behaviour, similar to the link between homophobia and self-destructive behaviour. In a personal account, the mother of a transwoman reports that her daughter (registered as male at birth, and 23 years old at the time of the writing of this paper) during her teens has seriously attempted suicide seven times, and in every case those attempts followed instances of humiliation (being spat at, the skirt being raised and similar incidents).^11^

It is thus possible that shame, as Goldberg suggested, may be the emotion that is *fed in* by social rejection and that *leads to* self-destruction. This may mean that it is not being gay or lesbian or transgender that is intolerable; and perhaps it may not even be social rejection per se that is intolerable: if Goldberg is right, it is *shame*.

This is important, because even the most sympathetic accounts of gender dysphoria often report how people with gender dysphoria often suffer concomitant anxiety and depression, which are usually regarded as *secondary to gender dysphoria* (that is, once the dysphoria is treated, anxiety and depression subside) (Megeri & Khoosal, 2007). If the analysis in this paper is accurate, it follows that this way of presenting the relationship between gender dysphoria and psychological health is only partially correct: presenting anxiety and depression as *secondary to gender dysphoria* covers up the social causes of anxiety, depression, and self-destructive behaviours; in other words, covers up the social determinants of shame. Insisting that anxiety and depression are *secondary to gender dysphoria* again results in a dangerous and myopic focus narrowly directed to the individual. Ill psychological health may not be ‘secondary to gender dysphoria’ at all; it may be that ill psychological health is the *direct result* of social rejection and the resulting shame.

Thus understanding shame (and perhaps guilt) should be central to understanding the complex experience of gender variance. Without proper consideration of these emotions, the predicaments of many transgender individuals cannot be understood and dealt with adequately.

## Transgender stories: am I feeling guilt or shame?

As we saw earlier, usually clinical psychologists maintain that there is a distinction between shame and guilt.

But whereas at times the emotion of shame is rather easily identifiable (remember the narration of Jess by Feinberg earlier – quite simply, Jess feels terrible shame), it can be at other times extremely difficult to differentiate shame from guilt. I will illustrate this difficulty with two examples. The first is an account provided by an anonymous source. The other is a song, which is based on a real story and co-written by the transwoman Fernanda Farias.

An anonymous source writes:

*[The problem with gender diversity is] not just about you, it is about those you love and the social environment you have to live in. It takes tremendous courage to wear your soul on your shoulders and go and show the ‘real’ you to the world. Even more so to your parents, who have dreams and aspirations that are in line with physical gender […] The fear of rejection, bullying, hatred are very real even with family for most of these children. The fear of total dismissal of how you feel can be even worse […] In the end you make a choice, you live a life where you face untold difficulty to be who you are and perhaps have to give everyone and everything up to achieve that. Or you live in the shadows never achieving peace and living a life that can never truly be fulfilled.*[12]

The woman here, registered as male at birth, decided to never transition, and thus to continue to live as a man, because she could not cope with the disappointment and hardship that she would have caused to her family. Having married and having had children as a man, she decided to ‘stick’ to her roles as a husband and father. This caused her enormous suffering. She now describes her life as a life in which *peace can never be achieved*, and in which *she can never find fulfilment*. She feels that hers is a life of constant deception, a masquerade, and a daily struggle. But revealing her female identity meant to her *betraying* the expectations and securities her family had built around him as a man.

There may be various reasons why she, or others in her situation, may decide not to transition (practical difficulties, medical concerns, concern for the welfare of the significant others) and shame and guilt may not be the only emotions or even the primary emotion at stake. It also ought to be recognised that not everyone will experience the type of problems that have been afflicting this particular woman. However, it is important to note that the predicaments that would typically be associated with shame (for example withdrawal, hiding) are not easily differentiated from those that are typically associated with guilt (feeling bad for specific acts). If one were to attempt to disentangle in this case which elements of her predicaments are ‘shame’ (that is, which are about the self) and which are ‘guilt’ (that is about specific behaviours) the task would prove arduous if not impossible; the woman here talks about being “who you are” and therefore about *her self* but also about specific behaviours (the roles she has as a husband and father). It is thus not entirely clear which parts of her predicaments are about *her self* and which are about specific actions. It is likewise difficult to differentiate which elements of the person’s predicaments are linked to social rejection (shame), which she mentions, and which are linked to the concern for others’ welfare (guilt). Finally, it is difficult to distinguish between hiding and reparative action: hiding, in this case, can be a *reparative* action. Hiding can be not just the desperate attempt to withdraw from the others’ awareness, but also a way to protect others, to keep them safe from shock or distress. Thus in order to understand the predicaments that may afflict transgender individuals one may need to be aware that each individual will be likely to experience transitioning in a unique way, and that the categories and classifications typically used in clinical psychology, however helpful, may in some cases not capture the complexities of people’s experiences.

Another emotion that seems to emerge in the narration above is the sense of betrayal. The woman here decides *not to betray* the expectations that others have built around her as a man, and thus lives a life that betrays only *her own self*. Because gender is about *identity*, it is about *who we are*, and who we are *for those around us*, transgender shame and transgender guilt may be closely intertwined.

A further example of the complexities of the relationship between shame and guilt in transgender individuals is illustrated in a song called *Princesa* and written by Fabrizio De Andre’ with Fernanda Farias (alias Princesa). The song is based on the real life of Fernanda, and thus provides an account (albeit poetical) of her own journey towards transition and of her own experiences (De Andre’, 1996). Here we can also see how deep the sense of betrayal can seep for transgender individuals.

Fernando was born male and had female gender identity since childhood. Being unable to transition in Brazil, Fernando moved to Italy as a clandestine immigrant and worked as a prostitute in Milan in order to raise money to pay privately for gender affirming surgery (Farias de Albuquerque & Jannelli, 1996). Fernanda, like many transgender individuals, was desperate to live a life congruent to the self and simply could not tolerate her male physique. However, in this poem, written after the transition to female, and so when Fernanda is finally living as a woman, Fernanda cries: “…*that Fernandino has died in my womb*”. Fernando is here Fernand-*ino*: the suffix ‘ino’ in Brazilian means *small, little*. This small child has died. Fernanda is responsible, perhaps guilty of this death: by creating (metaphorically) a womb (thus by transitioning), Fernanda kills her own self; her prior/other self is presented here (by Fernanda herself) as the innocent powerless ‘little Fernando’, who becomes in the metaphor her own child, the disempowered part of the self whose destiny is in the hands of Fernanda. Fernanda ‘creates’ herself (“Fernanda is a doll of silk” the song continues): Fernanda has to destroy Fernando in order to fulfil this creation.

Fernanda’s grief epitomises the experience of having to renounce one identity to embrace another. Fernanda is not of course the only person to mourn: she also eliminates Fernando from the lives of her father and mother, her siblings and everyone else who had been in Fernando’s life. Fernando will no longer exist and Fernanda is responsible for it.

It may be contended that nobody ‘chooses’ to be gender dysphoric, and thus that it is irrational for a transgender person to feel guilt. Yet it is the person who transitions who does in fact transition, who is the agent and the beneficiary of ‘the choice’. The transwoman cited earlier decided not to bear this responsibility and sacrificed her identity. Fernanda went on to seek alignment of her body and her life with her gender. Sadly she killed herself a few years after this song was released, in 2000.

These two stories illustrate how difficult it can be to differentiate shame and guilt in transgender individuals. It is almost impossible to disentangle which elements of the predicaments are ‘shame’ and which are ‘guilt’; which depend on the fear of social rejection, or on the fact of being socially rejected and vexed, and which depend on the preoccupation of upsetting meaningful others; it is also difficult to separate a way of coping that can be unequivocally regarded as ‘hiding’ as opposed to one that can be unequivocally regarded as ‘reparative action’. Hiding can be experienced as a way of protecting others, not just one self, and thus can be a reparative action.

There may be other complex issues afflicting transgender people, and of course not all transgender individuals will experience the torments expressed by Fernanda, but shame and guilt are likely to be central to the experience of many of them.

In the remainder of this paper I will try to draw from further literary work in the attempt to gain a better understanding of shame. I do this with the awareness that shame and guilt may not perhaps be always sharply differentiated in transgender individuals. However, attempting, albeit with a significant degree of approximation, to focus on shame has one important advantage, as anticipated earlier: it allows us to put into neat focus the social determinants of many of the predicaments suffered by transgender individuals.

## The poet of shame: Franz Kafka

Whereas shame has had a relatively marginal role in clinical psychology, at least until the 1960s, philosophers, artists and writers have been interested in it for centuries (Plato, 1970; Plato, 1974; Plato, 2002; Spinoza, 1947; Williams, 1994). Among all of them, there is one for whom shame has been so important as to be named “The poet of shame” (Friedlander, 2013): that is Franz Kafka. Kafka provides penetrating accounts of shame; but Kafka provides also an unsparing cross-section of human psychology: as Adorno wrote, Kafka is an “information bureau of the human condition” (Adorno, 1981).

Kafka has always been interested in shame. Walter Benjamin pointed out that shame in fact underlies probably all literary production by Kafka, and maybe his whole existence, and there could be various reasons for this (his difficult relationship with religion, his precarious health) (Benjamin, 1965). Kafka’s earliest novel was called “Shamefaced Lanky and Impure in Heart”: very little is known about this novel, as it was never published and is only known because Kafka mentioned it in a letter to his friend Oskar Pollack (Kafka, 1977). The story, as far as is known, juxtaposes two antithetic characters, the dissolute Impure and the moral Lanky. Lanky is the one who suffers pervasive shame.

Kafka is perhaps mostly famous for his short story *The Metamorphosis*, written probably earlier and published in 1915. I will discuss *The Metamorphosis* in the next section briefly, but the main focus will be on *The Trial*.

Neither *The Trial nor The Metamorphosis* have anything to do with transgender people (*The Metamorphosis* may, at first sight, appear more congruent with the theme of this article as it narrates about a man who wakes up in the body of a huge insect). However, particularly *The Trial*, as we are going to see shortly, provides a poignant account of how the experience of shame can be so insidious as to be easily unnoticed until its devastating consequences are irreparable (the reader, in fact, is unlikely to think about shame until the last few words of the novel).

### The Trial

*“Like a Dog!” – he said - “It was as if the shame of it should outlive him”* (Kafka, 1999, p. 128).

*The Trial* finishes with the announcement of the *survival of shame*. In this novel, written between 1914 and 1915, and published in 1925, we find a portrait of shame that is extraordinarily sophisticated, and sheds good light into the psychological dynamics of shame.

Josef K is employed by a bank. One morning, two unknown men arrest him and Josef K realises that he needs to stand a trial for some crimes of which he is not made aware. Josef K at some point, completely disempowered, refuses to defend himself. He cannot tolerate the lack of transparency that characterises the whole trial. The tribunal is described in various places as lurid. The Judge’s books are dirty, dusty – this is what they study and these are the people who will judge him. Josef K refuses thus to defend himself, and this sanctions his own end. He is led in a cave where he is executed, stabbed twice in the heart.

The atmosphere of the novel is anguishing, and Josef K somehow surrenders to an absent guilt - a guilt that is attributed to him, though it is not clear of what he is guilty or whether he is guilty of anything at all. He is condemned and will be executed for a crime that he has probably not committed, but he surrenders nonetheless. The psychological portrait here of a man trapped by the others’ accusations, even in the full knowledge of their absurdity, is poignant and painful. Logic would suggest that knowing that there is nothing to be guilty of, one would defend oneself or attempt to escape. But Kafka seemed to know well that the way humans respond to what happens to them often escapes logic – and yet it is possible to understand Josef K’s behaviour – indeed this possibility of identifying with the protagonist contributes to the disquiet of reading. The maze of threats, false accusations, innuendos, false hopes, opaque promises, which is constructed around Josef K is so tight that, psychologically, he remains trapped in it.

It may be also noted that he is Josef *K*, not simply Josef. There is a mysterious element to Josef K, an enigma *hidden out on the surface*, on his very name. We are introduced to him as an ordinary man; yet his name remains hidden, but hidden, I propose, out on the surface, as we are made conscious of the fact that he has a surname that is being deliberately omitted.

Josef K - not less enigmatic than the thread of which he is a prey – thus curiously makes no attempt to defend himself against the two men who lead him to his grave. He takes off his own shirt and lies down to be executed.

While Josef K is stabbed to death, he says: “Like a dog!”. He is killed like a dog: “It was as if the shame of it should outlive him” (Kafka, 1999).

The extraordinarily acute end of the episode reveals Kafka’s sharp psychological insight into human nature. *Shame* cleverly underlies, without being mentioned until the end, the entire novel (Wasihun, 2015): it is because of shame that Josef K surrenders, not because of guilt. The whole apparatus constructed around him does not at any stage convince him of his guilt: at no point Josef K wonders whether he really committed some kind of crime. Nonetheless (perhaps *because* of this) he voluntarily surrenders. Josef K has his own obscure side. Thus, ultimately, there is no point in fighting because the accusations can be false and not false at the same time.

As a commentator has pointed out, the shame of Josef K “is a shame that looks for its guilt and that, failing to find it, foments itself” (Latini, 2012a). It is a similar shame, as we are going to see in what follows, which may afflict transgender individuals. A shame that touches one of the most intimate aspects of one’s self, namely gender, one of the most fundamental segments of our identity, and a shame that is the effect of a lack of humanity in *the other*, and which, precisely for that reason, is inescapable.

As we saw earlier, clinical studies have suggested, from quite a different perspective to that adopted by Kafka, that there is no reparation in shame (whereas there is possible reparation in guilt). Once we are ashamed, the experience may be hard or impossible to reverse. Kafka saw very clearly and piercingly how others can disempower us to the extent of self-destruction - even more so when there is no ‘real’ or objective fault of our own.

### The Metamorphosis: the plot

Perhaps Kafka’s most famous novel is *The Metamorphosis*. This novel is a genuine story of *trans-genderism* – literally meant as *going beyond birth*. As in *The Trial* we are introduced to an ordinary man, Gregor Samsa, who wakes up in the body of a huge insect. The way in which Kafka unfolds the thread provides another unflattering cross-section of human psychology.

When his mother knocks on the door to check why he is not going to work, Gregor hides himself: he feels shame. The more attention he gets (his sister is worried, his employer shows up to check on him) the more shame he feels.

Kafka here (as in other novels) makes an implicit deal with the reader: the reader has to be willing to accept a paradox. Once the paradox is accepted, the story becomes painfully realistic. Gregor’s employer threatens to fire him, as he persists in refusing to open the door and to provide a sensible explanation for his behaviour. The employer’s lack of faith in Gregor is evident: this leads him to make threats that are completely inappropriate to the circumstances, and such disproportion renders him and his menace grotesque. Once Gregor manages to open the door, the acknowledgment he gets is far from favourable. The father throws him back into his room hitting him with a newspaper and banging his feet on the floor to scare him, the employer runs away in disgust, the mother faints.

Gregor tries to live as an insect in his room. He enjoys crawling up and down the furniture; his sister feeds him, and he hides in order not to frighten her. The hiding here – similarly to the case of the transwoman mentioned earlier - is not just *withdrawal*, but is reparative, and reparative of a guilt which is at once material and inexistent– a paradox also well illustrated in *The Trial*.

Gregor’s family does not accept him as an insect. Being rejected, unloved, and feeling he is only a burden to his family, Gregor falls in a deep depression and stops eating, and condemns himself to a slow death (the ultimate reparation, and the ultimate withdrawal).

His death is somehow ironically prosocial: after his death, things improve for his family. They soon forget the unfortunate episode, and all goes back to normal.

## Shame in transgender individuals: a social issue

What Kafka inflicts to his characters both in *The Trial* and in the *Metamorphosis* is (among other things) shame. Shame may relate to the incommensurability of one’s experience with the experiences, assumptions and expectations that significant others may have of us (as in Kafka’s *Metamorphosis*). But there is more: because gender is an ‘essential’ segment of one’s identity, the process of shaming someone because of their gender identity can go as far and as deep as to put into question their very status as *persons* (Butler, 1999).

Not belonging to a gender questions the very status of who we are, indeed, whether we are anyone at all.

In *Gender Trouble*, Judith Butler wrote that if sex and gender are assumed to be stable and immutable, then the identity of a person is a function of them (Butler, 1999). Those who violate the norm, then, are not just different or part of a minority: their status as ‘persons’ is in question. As I have pointed out elsewhere, the case of transgender people is in this sense similar to the case of nomads. They are not in the State but are not outside the State. They are not citizens but are not foreigners either. They are inhabitants of no man’s land. This not belonging to the only two categories that are accepted by organized States renders nomads culturally unintelligible. Transgender individuals may suffer the same form of cultural unintelligibility (or intelligibility on the condition of being understood as ill) (Giordano, 2011).

The threat, in these cases, is to be placed at the borders of humankind (Giordano, 2013c). It is when people are placed at the borders of humankind that they can more easily be divested of their protective dignity and mostly exposed to violence (Arendt, 1964; Glover, 2012).

Latini makes an interesting observation on Kafka, which can tell us something important about transgender shame: the shame that underlies *The Trial* is the shame *of an intimacy that becomes of public domain* (Latini, 2012b). Both *The Trial* and *The Metamorphosis* begin with a private, intimate act – the act of waking up and getting ready for the day. The intimacy of these ordinary first moments of the day is violated, and this is the first dimension of the shame experienced by the two protagonists. Similarly, in *The Trial*, the intimacy *of death* is violated by the two executioners; cheek-to-cheek they check that the blade, twisted twice in Josef K’s ribcage, has produced the desired effect, and Josef K, dying under the gaze of these two executioners, feels shame, literally *limitless* shame, *unlimited* shame, not delimited by death, and not even limited by the awareness of being a victim of the greatest injustice.

Primo Levi pointed out another interesting aspect of shame, which again can tell us something important about the experience of shame in transgender individuals. Levi, a Jewish chemist and writer and Holocaust survivor, translated *The Trial* into Italian (1983). Levi described his work on Kafka’s *Trial* as painful to the extent that, at the end of it, it seemed to him to have overcome a terrible illness. Levi saw in the shame of Josef K the shame of all those victims, like himself, who are prosecuted and condemned for crimes that they have not committed (Levi, 2009a). Of a similar feeling Levi talks in another book, *Sommersi e I salvati*, (*The Drowned and the Saved*). In this book he described the moment in which Russian soldiers approached the Lager during the liberation, and in that moment, Levi says, they appeared to feel *shame*.

They did not greet, did not smile; they appeared oppressed, not only by pity, but also by a confused restraint that sealed their mouths […] It was the same shame which we knew well, in which we drowned after each selection, and every time we had to witness or withstand an offence: the shame that the Germans did not know, which the righteous person experiences in front of crime committed by the unjust, the crime whose existence stings, *for its being introduced irrevocably in the world of the things that exist, the crime against which one’s will has been able to do nothing or next to nothing; the crime against which one’s will has produced no defence* (Levi, 2009b).

Levi, thus, in his tragic experience of the holocaust, noted that shame can be just about *the guilt of others*. We may feel shame just for *sharing* the same humaneness of those who irrevocably introduce crimes into the “world of the things that exist”.

Other writers have been interested in the shame experienced by survivors of concentration camps (Agamben, 1998; Anders, 1992). I do not wish of course to compare the experience of shame in transgender individuals with the experience of shame in survivors of various crimes and war crimes; but Agamben’s and Anders’s considerations may be pertinent here: they both note, similarly to Levi, that the *victims’ shame* parallels *the guilt of the executor*. The shame that survives Josef K is, as mentioned earlier, the shame for a crime *not committed*, the shame for *one’s innocence,* but for that reason also the shame *for the dis-humanity of others*.

This double dis-humanisation may be one of the keys for understanding the experience of shame in transgender individuals. In the process of shaming, particularly when this is experienced again and again, transgender individuals are not only divested of their protective dignity, de-humanised, and thus further exposed to violence; they are not only placed quite literally at the borders of human-kind (a human-kind jealously attached to binary ideas of sex and gender and that thus still struggle to fully legitimise the very ambiguity of the notion of gender) but they, like a mirror, reflect the dis-humanity of others, they highlight it or even bring it into existence.

Shame is not thus just an obvious response to humiliation; and it is not only a personal reaction, but, as Benjamin also pointed out, is also a socially determined reaction, and a socially “demanding reaction” (Benjamin, 1965).

## How do people cope?

There is paucity of systematic studies of transgender coping with shame; but one insightful piece of research evaluated how young LGB people cope with homophobia, and showed how all the coping mechanisms implemented were, effectively, *shame-avoidance* strategies.

This research has, as its main focus, sexual orientation in adolescents and young adults (not gender dysphoria), but what this research tells us is relevant to understanding shame and its social and political nature in transgender individuals (Mcdermott et al., 2008c). The researchers found that young people cope with homophobia in mainly three ways: minimisation, responsibility and creation proud identities.

‘Minimisation’ refers to the ‘normalisation’ of cruelty. See the following extracts:

“Jane: ... I got bottled actually in a homophobic attack and, eh, ended up in hospital a few years ago which is nothing you know really serious, plenty of people get bottled anyway ... (laughs) (gay, 25, South Wales)

Cherie: I used to get beaten up on the way back and like, are you a boy as well? The usual stuff ... (lesbian, 17 North West of England)” (Mcdermott et al., 2008c).

Cherie talks about ‘the usual stuff’; she presents abuse as routine; similarly Jane describes her attacks as commonplace. In this way, homophobia is constructed as mundane, as a fact of life. This, according to the researchers, is a process implicitly meant to reduce the significance of the abuse, and to deflect the shaming effects of homophobia (Mcdermott et al., 2008c).

The second modality of coping is ‘taking responsibility’, being an adult. See the following extract:

“Deborah: I’m not 10 years old anymore I’m 16, I’ve grown up and I’m leaving home soon so just get over the fact that I am what I am, that I like girls as well as lads, and just say, tell him, ‘fuck off’. (Bisexual, 16)” (Mcdermott et al., 2008d).

The final modality of coping is the ‘creation of proud identities’. The researchers found that their participants’ reports included comments such as “he is fine in himself and does not care about what other people think”: the idea here is that being comfortable about who one is protects the individual from other people’s disapproval.

All these modalities are, according to the researchers, *shame-avoidance strategies*. They are clearly important: individuals can develop resilience against other people’s implicit or overt rejection. In other words, without necessarily pretending to eradicate overnight homophobia and transphobia, people can learn to reduce their shaming effect on them. However, as the researchers acutely observe, any and all these strategies are *individual*; they depend on the individual’s abilities and resilience. All of these strategies may thus dissuade sufferers from seeking help, because seeking help may again turn the “shaming-spotlight of homophobia” (Mcdermott et al., 2008d) towards the individual.

The danger is that individual resilience may exacerbate the risk of self-destructive behaviour in transgender individuals and other vulnerable individuals. Individual empowerment functions on the idea that there are ‘those who can cope’ (the proud, the resilient, those who can ‘hold their head high’) and ‘those who cannot’ (those sensitive to shame, who are distressed by the social responses, who shy and hide away). But being resilient is easier in some contexts and harder in others, easier at some times in our lives and harder at others, not *just* easier for *some individuals* and harder for *others* (as in a dichotomy of ‘strong’ and ‘weak’).

It may be useful here to draw some further insight from narrative. In the *Cyrano de Bergerac*, Rostand gives us a beautiful example of someone who suffers extreme shame, and who learns to hold his head up high – and we see here the circularity of shame and pride.

## The big nose of Cyrano de Bergerac – pride and shame

*Cyrano* was a real French cadet and writer who lived in the 1600s. Rostand romances Cyrano’s story and attributes to him an enormous nose. Cyrano is as capable with his words as he is with his sword; he compensates his unusual look with his extraordinary wit and dexterity. But when it comes to his love life, his nose is in the way (psychologically as well as perhaps literally).

Cyrano falls in love with Roxanne, but is so ashamed of his nose that he decides not to declare his love. He goes further: he drives her in the arms of the good-looking but rather inept Christian. He writes verses for Christian to recite to her. Cyrano hides his face behind the (in his eyes) acceptable persona of Christian. He obtains a meagre consolation: Roxanne indeed falls in love with Cyrano, mistakenly thinking she is in love with Christian. She falls in love with Cyrano’s thoughts and feelings, of which Christian is the hollow spokesperson. It is hard to think of a more painful example of shame and withdrawal than the one chosen by Cyrano.

But somewhat paradoxically, this enormous nose is also at the source of great *amour-propre*: In Act I Cyrano chants the pride of his nose before other cadets. He becomes sarcastic and nearly verbally aggressive:

*Who find my visage's center ornament*

*A thing to jest at--that it is my wont—*

*An if the jester's noble--ere we part*

*To let him taste my steel, and not my boot!*

Cirano jokes about his nose, with *pride*:

*'When you sup*

*It must annoy you, dipping in your cup;*

*You need a drinking-bowl of special shape!'*

*Descriptive: “Tis a rock!. . .a peak!. . .a cape!”*

*--A cape, forsooth! 'Tis a peninsular!* (Rostand, 2010)

This is of course the same Cyrano who is so ashamed about his nose as to renounce love.

Cyrano reminds us that resilience and pride can be the other face of painful and persisting shame. Moreover, he reminds us that resilience and pride have to be negotiated and maneuvered in different situations and at different times, and there is no such a thing as the strong individual who is capable of resilience and the weak sensitive one, who is victimized.

Resilience and pride require constant negotiation, especially in individuals who have experienced social rejection and abuse. But this negotiation should not rely only on individual skills: social support must be a part of the process. Empowerment should not be an aim on its own: social (and political) action is needed concomitantly, to reduce shame.

## A few final thoughts and recommendations

This paper gestures towards a number of conclusions and recommendations (clinical and socio-political).

First, understanding shame should be central to understanding the complex experience of gender variance. This paper calls for a systematic study of shame in transgender individuals and communities, and suggests that other disciplines may provide valuable insight into aspects of shame that may defy clinical observation, as they may not be easily captured by the current paradigms used in clinical psychology^13^ (Kuhn, 1996).

Second, shame is not only, or even primarily, an intra-pscychic issues. Shame, as illustrated by Josef K, and as we have seen in the case of war crime survivors, stems deeply from the awareness of the dis-humanity of our fellow humans; it is the awareness that something horrible has been irrevocably placed, as Levi put it, “in the world of the things that exist”.

This has important social (and possibly political) implications. Shame cannot be addressed solely by addressing people’s resilience; it has to be tackled with appropriate policies and law that reduce still pervasive homophobia and transphobia. But this also has clinical implications: the job of the therapist involved in understanding and helping people to cope with shame somehow risks missing the social causes of shame. In other words, in helping the individual to elaborate and become more resilient to the emotion of shame, the therapist risks inadvertently to de-legitimise that emotion, which in certain circumstances, even when it persists over time, is arguably an adequate response to persisting social rejection and abuse. In turn, this raises questions as to what the role of the therapists should be in providing help to people affected by various forms of trauma, minority stress and abuse. It becomes unclear, from this perspective, whether the work ‘with the individual’ in the individual encounter between patient and therapist can be effective, or may instead risk being deleterious, as highlighted in “How do people cope?” section; and indeed whether it is morally justified at all, when the causes of the distress are not primarily intra-psychic. Of course this is not to suggest that therapists’ practice is immoral and that patients ought to stop seeking support, but it is to invite a reflection on the legitimate aims of therapeutic work.

Third, we have seen, particularly through the narration of Josef K’s execution, that the experience of shame can be insidious, and can go unnoticed until its devastating consequences are irreparable (it is interesting that the reader of *The Trial* is unlikely to think about shame until the last few words of the novel). As *The Trial* illustrates vividly, and as clinical research discussed here shows, people who experience shame are prone to surrender (like Josef K and Gregor Samsa), and may either accept abuse, or expose themselves to abuse, or adopt other forms of self-destructive behaviour. The third recommendation, thus, is to be alert to the possibility of shame, even when that emotion is not clearly or obviously displayed or verbalised.

Finally, gender is one of the most fundamental segments of our identity: most of us identify as a woman, or a man, or somewhere along the woman-to-man spectrum (perhaps even the gender neutrality of a-gendered individuals is a form of identity). Questions about our gender are in essence questions about who we are as persons, and thus being ashamed about our gender is also an attack to our status as persons. As we have also seen in the case of Gregor Samsa particularly (but also of Josef K and again of war crime survivors), shame appears at times because of a violation of intimacy – our intimacy becoming of public domain. Gregor Samsa’s saga is related to his different bodily appearance: for that reason he is no longer recognised as a member of his family, as a person worthy of concern. Gender, like Gregor’s appearance, cannot be purely a private matter – it has an obvious public dimension. Yet it is also a very personal and intimate matter. We have seen in this paper that when something so intimate, and so, literally speaking, *essential* to one’s identity becomes (by necessity) of public domain, this is likely to expose people concerned to shaming experiences and to the associated risks.

The clinical importance of shame cannot be underestimated, considering the serious perils associated with it. But shame is also important because it exists in a context, not just in a mind. Shame is not the obvious response to humiliation, and it is not only a personal reaction: it is a socially determined reaction. Shame is likely to tell us something about the social environment that hosts the individual. Shame has a social nature, particularly in the case of gender variance: the experience of shame is a function of how gender and transgender identities are construed, recognised, accepted, validated or rather vexed in certain socio-cultural contexts.

## Footnotes

1 The term ‘narrative’ comes from the Latin *narrativus* which means ‘telling a story’. The word ‘narrative’ here refers therefore broadly to every account, be it biographical or fictional. However, to avoid confusion between biographical account and literary works, wherever possible and appropriate, I will refer to personal accounts as ‘narrations’, or ‘personal reports’. It must be noted, however, that in the conclusion I will refer to the work of Mia Siflver, who uses the words ‘narratives’, ‘narrative’, ‘narrations’ very broadly to encompass personal accounts as well as literary works. See (Silfver M. 2007).

2 A picture of the artwork can be found here https://thesmartset.com/article12041201/

3 A picture of the artwork can be found here https://uk.pinterest.com/pin/552746554241491998/

4 One exception is Wallace R, Russell H 2013. Here the authors note the link between shame and gender identity in children with gender dysphoria. They also connect shame with attachment theory. However, they focus on the philosophical and clinical approaches by Zucker and Menvielle, which are diametrically opposing approaches to gender. Zucker regards gender dysphoria as pathology and, under this perspective, successful therapy is that which conducts the child to align to the gender assigned at birth. Menvielle does not regard gender dysphoria as pathology and under this perspective therapy succeeds when the child is able to adjust to whatever gender she or he has. Wallace and Russell attempt to propose a third framework that surpasses such dichotomy, based on prevalent attachment theories.

5 Among the first psychologists to have studied shame as differentiated from guilt we should mention Silvan Tomkins.

6 It should be noted that by that time, in cultural anthropology this distinction was already accepted. Eric Robertson Dodds, in 1951, published a book called *The Greeks and the Irrational*, in which he coined the distinction between ‘guilt’ cultures, that is societies in which social control is achieved through inducement to guilt, and ‘shame’ cultures, that is societies in which social control is achieved through inculcation of concepts of honour and pride.

7 A picture of the artwork can be found here http://isoladipatmos.com/wp-content/uploads/2014/10/5154455318_73e0314c8a.jpg

8 A picture of the artwork can be found here https://it.wikipedia.org/wiki/Ebbrezza_di_No%C3%A8#/media/File:Ivresse_de_noe.jpg

9 The Genesis tells this story: *20 In those days Noah became a farmer, and he made a vine-garden. 21 And he took of the wine of it and was overcome by drink; and he was uncovered in his tent. 22 And Ham, the father of Canaan, saw his father unclothed, and gave news of it to his two brothers outside. 23 And Shem and Japheth took a robe, and putting it on their backs went in with their faces turned away, and put it over their father so that they might not see him unclothed.* See Genesis in the reference list.

10 A picture of the artwork can be found here http://www.museosansevero.it/pudicizia/

11 Broadcasted on BBC on 12 September 2017, at http://www.bbc.co.uk/programmes/b0930znz

12 Anonymous, personal communication.

13 It may be worth noting that the epistemic limits of the paradigms used in any science has long been debated in philosophy of science. One of the first philosophers of science to have discussed the problematic nature of scientific paradigms is Thomas Kuhn, in his *The Structure of Scientific Revolutions* first published in 1962.
